# Facilitating Personal Recovery in Individuals With Mental Illness: A Longitudinal Qualitative Interview Study of a Person‐Centred Group Intervention

**DOI:** 10.1111/inm.70329

**Published:** 2026-08-13

**Authors:** Kattie Legaard Hostrup, Kira Jensen, Flavia Maria Maties, Rikke Jørgensen

**Affiliations:** ^1^ Psychiatric Department Aalborg University Hospital – Psychiatry Aalborg Denmark; ^2^ Department of Clinical Medicine Aalborg University Aalborg Denmark; ^3^ Unit for Psychiatric Research Aalborg University Hospital – Psychiatry Aalborg Denmark; ^4^ Department of Clinical Medicine Aarhus University Aarhus Denmark; ^5^ Psychosis Research Unit Aarhus University Hospital, Psychiatry Aarhus Denmark

**Keywords:** CHIME, group intervention, mental illness, patient centred care, recovery

## Abstract

This study explored how participation in a person‐centred group intervention influenced the personal recovery process of individuals with mental illness at the end of the intervention and 3 months after. Using a qualitative longitudinal design, the study was conducted in an outpatient clinic at the Psychiatric Department of the North Denmark Region. Seventeen participants completed the 12‐week intervention, which included two Discovery group sessions and 10 Guided Self‐Determination method sessions. Participants were interviewed post‐intervention and at three‐month follow‐up. Data were analysed using reflexive thematic analysis by Braun and Clarke and the CHIME framework with a deductive approach. The intervention positively influenced participants' personal recovery process across all CHIME categories. Being among peers fostered a sense of belonging, trust and mutual understanding leading to greater openness and confidence in relationships within and beyond mental health services. Participation in the intervention enhanced self‐understanding, hope and optimism about the future, though some participants emphasized the need for ongoing support to sustain these gains. Engagement in the group promoted self‐acceptance and openness about mental illness, increased awareness of factors affecting well‐being and encouraged focus on opportunities and quality of life. Participants also reported greater self‐reflection, self‐esteem and responsibility, leading to a stronger sense of control over their lives. Overall, the person‐centred group intervention supported participants' personal recovery process by strengthening connectedness, hope, identity, meaning and empowerment. Continued professional support was viewed as essential to sustain and integrate these positive changes.

## 1 Introduction

Person‐centred care (PCC) involves healthcare providers collaborating with patients to design and deliver care tailored to the individual's specific needs and preferences (Santana et al. 2018). PCC is widely recognized as a key approach in mental health services, contributing to improved health outcomes and enhanced patient well‐being (International Council of Nurses 2024). Effective implementation of PCC supports the delivery of care that is responsive to individuals' unique needs and circumstances (International Council of Nurses 2024). Moreover, PCC contributes to improved healthcare quality and patient safety by ensuring that care is guided by each person's preferences, needs and values (Kruk et al. 2018; World Health Organization, World Bank Group and OECD 2018).

## 2 Background

Closely aligned with PCC principles, recovery‐oriented care further emphasizes individuals' autonomy, resources, and desires to achieve a meaningful and hopeful life, and health professionals forming relationships based on trust, being hopeful for the individual's future and respecting the individuals' experiences and knowledge from their own life (Jørgensen et al. 2022). Among the most common person‐centred care approaches are recovery‐oriented models and frameworks (International Council of Nurses 2024; Bjørlykhaug et al. 2022).

One key concept within this is personal recovery, often described as a unique, individual process through which people learn from their experiences and develop skills to pursue meaningful goals in life (Anthony 1993). Rather than reflecting linear symptom‐based outcomes, personal recovery is increasingly understood as a multidimensional and non‐linear process shaped by individuals' relationships, social roles, environments and sense of self (Van Weeghel et al. 2019). The personal recovery process has been operationalized in a systematic review providing a theoretical framework comprising five recovery processes described as five categories with subcategories: Connectedness (support from others and being part of the community), Hope (a positive view on the future and motivation to change), Identity (building a positive identity and overcoming stigma), Meaning (developing meaningful roles and activities, quality of life and spirituality), and Empowerment (gaining control over life and focus upon strengths), giving the acronym CHIME (Leamy et al. 2011). The CHIME framework was originally developed from studies primarily involving individuals with psychotic disorders (Leamy et al. 2011), but has since been applied across a range of diagnostic groups, including depression (Richardson and Barkham 2020), eating disorders (Wetzler et al. 2020), bipolar disorder (Jagfeld et al. 2021), autism (Lases et al. 2025) and substance use disorders (Dekkers et al. 2020). These findings suggest that the framework has transdiagnostic relevance, although variations exist in how its dimensions are expressed and prioritized across populations (Lases et al. 2025).

In recent years, there has been a shift from a primary focus on psychopathology toward personal recovery as an important complement to traditional outcome measures in mental health care and treatment (Maas et al. 2024). Personal recovery emphasizes living a meaningful and satisfying life despite the limitations associated with psychiatric conditions. Reflecting this shift, the CHIME framework has been widely adopted as an analytical framework in both qualitative (Eklund and Argentzell 2025) and quantitative studies (Moeller et al. 2024) examining processes of personal recovery. By capturing key dimensions of personal recovery, the CHIME framework offers a humanistic and experience‐centred lens through which care practices can be evaluated and guided, ensuring that interventions not only address symptoms but also promote autonomy, meaningful relationships and a renewed sense of self (Mutschler et al. 2022; Lim et al. 2020).

The person‐centred, evidence‐based intervention the Guided Self‐Determination (GSD) method has been applied across a range of health conditions, including diabetes, stroke, chronic pain, schizophrenia and ADHD, and has been well accepted by both patients and healthcare professionals, demonstrating value across diverse healthcare contexts (Linnet and Jørgensen 2023). The purpose of the GSD method is to support individuals in managing their illness by supporting them identify everyday challenges, explore possible solutions, and strengthen self‐management skills (Zoffmann 2004). The GSD method aligns with a recovery‐oriented approach by prioritizing individuals' experiences and understanding of their own challenges rather than positioning health professionals as primary problem‐solvers (Jørgensen et al. 2012). Furthermore, the GSD method has been described to impact personal recovery (Jørgensen et al. 2015), as measured by the Recovery Assessment Scale (Corrigan et al. 1999). The GSD method has been delivered in both individual and group formats (Linnet and Jørgensen 2023) however, its application in a group format within mental health services has not yet been evaluated. Group‐based treatment and care are well established in mental health outpatient settings (Caruso et al. 2013) providing a relevant context for such an adaptation. In tailoring the intervention, two individuals with lived experiences contributed to the development of the group format and recommended that the initial two sessions be conducted as Discovery Group sessions. These sessions aim to provide a supportive environment in which participants can engage in dialogue with peers and mental health professionals (Barker and Buchanan‐Barker 2005), prior to commencing the GSD group sessions. The Discovery Group is grounded in the Tidal Model, a philosophical approach that is person‐centred and recovery‐oriented and emphasizes individuals' lived experiences, strength and active participation in their own recovery processes (Barker and Buchanan‐Barker 2005). Given that both the GSD method and the Discovery Group are grounded in a recovery‐oriented approach, and that the GSD method has not previously been evaluated in a group format within mental health services, an evaluation focusing on personal recovery is warranted. Although the Recovery Assessment Scale (RAS) captures several key aspects of personal recovery, particularly connectedness, hope and empowerment, it does not encompass the full range of experiential and narrative dimensions central to recovery as conceptualized in the CHIME framework (Pelgrim et al. 2026). In addition, the Tidal Model underpinning the Discovery Group aligns closely with core CHIME domains, including connectedness, empowerment and identity (Morén et al. 2026). Therefore, the CHIME framework was selected as the analytical framework to enable a more comprehensive assessment of the recovery‐oriented processes. Guided by the CHIME framework and a focus on personal recovery, the aim of the present study was to explore how participation in a person‐centred group intervention influenced the personal recovery process of individuals with mental illness, both at the end of the intervention and at three‐month follow‐up.

## 3 Methods

### 3.1 Design

This study is part of a larger mixed‐methods study evaluating both implementation outcomes and clinical outcomes of the PCGI (Jensen et al. 2024; Jørgensen et al. 2025; Benzon and Jørgensen 2024). It was designed as a qualitative longitudinal study based on individual interviews (Audulv et al. 2022). This design is consistent with a pragmatic epistemological stance (Kaushik and Walsh 2019), as it emphasizes practical and actionable outcomes, aiming not only to generate theoretical insights but also to optimize the PCGI for a more effective real‐world implementation. The study adheres to the Standards for Reporting Qualitative Research (O'Brien et al. 2014).

### 3.2 Setting

The study was conducted in 2019–2020 at three public outpatient clinics at the Psychiatric Department in the North Denmark Region, specializing in the treatment of individuals with affective and psychotic disorders. All clinics provide care and treatment for adults aged 18 and above and employ psychiatrists, psychologists, social workers and registered nurses. The PCGI intervention took place in the outpatient clinic at the same site as the participants went for ordinary treatment.

### 3.3 Recruitment

To be eligible for the study, participants needed to receive treatment at one of the three outpatient clinics and capable of providing informed consent. Recruitment of potential participants relied on convenience sampling (Emerson 2015), as individuals were approached during their appointments at the outpatient clinics either by a PCGI facilitator or their primary clinician. They were then invited to attend an individual information meeting about the research project. Individuals who agreed to participate received comprehensive oral and written information about the project and were given time to carefully consider participation. Upon signing informed consent, the participants were allocated to a group.

### 3.4 Participants

A total of 17 participants completed the PCGI, and 16 participated in the interview at the end of the intervention. The sample included seven females and nine males diagnosed with psychotic or affective disorders, some with alcohol dependency, and had a mean age of 37.3 years and an average of three prior hospital admissions. One participant did not show for the interview and did not respond to contact. At the three‐month follow‐up, interviews were conducted with seven participants, as the rest did not respond to the interview invitation. The participants' demographics did not differ from those of the larger sample counting three females and four males with psychotic or affective disorders, some with alcohol dependency, and had a mean age of 36.1 years and an average of three prior hospital admissions.

### 3.5 The Person‐Centred Group Intervention

The PCGI consisted of 12 weekly sessions with two sessions of Discovery group (Barker and Buchanan‐Barker 2005) and 10 sessions of the GSD method (Zoffmann 2004). The participants had a mean attendance rate of 73.33% (range 63.15%–91.30%). Each session involved one facilitator, 3–4 participants and lasted 75 min. The PCGI was conducted twice in 2019, facilitated by four experienced mental health nurses, three with postgraduate qualifications, all with over 10 years' experience, and prior group and individual GSD facilitation. All PCGI facilitators received supervision during the delivery of the intervention, which has been described elsewhere (Jørgensen et al. 2025).

#### 3.5.1 Discovery Group

The discovery group is developed within the recovery model, the Tidal Model (Barker and Buchanan‐Barker 2005). Its primary aim is to support participants to build their capacity to share with others through simple questions and answers. Participants take turns drawing pre‐prepared question cards and answering them in front of the group. This structure fosters meaningful reflection and encourages connections among participants (Barker and Buchanan‐Barker 2005). Facilitator and participant experiences with the discovery group are reported elsewhere (Benzon and Jørgensen 2024).

#### 3.5.2 The Guided Self‐Determination Method

The GSD method is a person‐centred intervention developed over 20 years ago (Zoffmann 2004). GSD involves the use of semi‐structured reflection sheets, which patients complete and bring to sessions with a health professional. The reflection sheets are structured into four topics: (1) agreement to work together, (2) your life with an illness, (3) between ideal and reality, and (4) working to change. The GSD sessions focus on facilitators using advanced communication techniques, such as active listening, mirroring and value clarification, to promote patient reflection, shared insight and collaborative problem‐solving (Zoffmann and Kirkevold 2012).

In the PCGI the GSD reflection sheets were divided into 10 sessions: (1) Lifeline and Difficulties in everyday life, (2) Unfinished sentences, (3) Picture or metaphor, and Room for mental illness in everyday life, (4) Recommendations for everyday life with mental illness, (5) Your reality with mental illness, (6) Experiences with treatment, and Identification of a current problem, (7) Problem‐solving, how have you solved the problem until now, (8) Problem‐solving, future ideas for problem‐solving, (9) Follow up on problem‐solving, (10) How to carry on from here.

Further information of the content, form and structure of the PCGI is published elsewhere (Jensen et al. 2024).

### 3.6 Qualitative Individual Interviews

Individual qualitative interviews were conducted at the end of the PCGI and 3 months after. The interviews were conducted by three clinical nurse specialists who were not familiar with any of the participants. All interviews were based on a semi‐structured interview guide (Green and Thorogood 2018) with interview questions such as: *Have you experienced handling challenges differently now compared to before participating in the PCGI?* and *What impact has participation in the PCGI had on your everyday life?*, with elaborative questions and follow‐up questions to gain a more profound insight into the participants' experiences and perspectives. The interview guide was pilot tested, which only resulted in minor adjustments. As the interviews were originally conducted as part of a larger research project, CHIME did not inform the development of the interview guide or the data collection process. Instead, the framework was introduced during the analytic phase to explore how participants' experiences could be understood in relation to personal recovery. Interview duration ranged from 14 to 38 min, averaging at 25 min. All interviews were recorded and subsequently transcribed verbatim.

### 3.7 Research Team

The research team consisted of four nurses: R.J. with a PhD employed as a senior researcher, and K.L.H., K.J. and F.M.M. with a master's degree employed as clinical nurse specialists in the Psychiatric Department in the North Denmark Region. Each team member had prior experience in qualitative research. None of the researchers had prior knowledge of or a relationship with the participants in the study.

### 3.8 Analysis

The data were analysed using theory‐driven reflexive thematic analysis by Braun and Clarke (2006, 2022). Thematic analysis was considered appropriate as it enables the systematic identification, organization and interpretation of patterns of meaning across the dataset. A deductive coding approach was applied, where data were coded in relation to the five CHIME domains and the subcategories, which conceptualizes personal recovery as comprising Connectedness, Hope, Identity, Meaning and Empowerment (Leamy et al. 2011). This approach allowed for a structured reflexive exploration of how participants' experiences reflected different dimensions of personal recovery.

In line with Braun and Clarke's reflexive understanding of thematic analysis, themes were not treated as passively emerging from the data, but as actively developed through the researchers' interpretive engagement with the material. This approach allowed for a theoretically informed yet flexible analysis of participants' accounts.

Initially, familiarity with the interviews was achieved through repeated reading and listening to the recordings. Initial codes were then generated across the dataset, guided by categories and subcategories from the CHIME framework while remaining attentive to data that extended beyond predefined categories. A matrix was then constructed to ensure an overview and maintain consistency throughout the analytical process. See Table 1 for the matrix illustrating the analytical process for the CHIME domain ‘Hope’.

**TABLE 1:** Analytic matrix illustrating the CHIME domain Hope: Example of the deductive coding process.

CHIME domain	CHIME subcategory	Illustrative quotes (selected)	Illustrative quoted—follow up (selected)	Condensed meaning	Codes	Analytic interpretation	Overall analytic interpretation
Hope and optimism about the future	Belief in the possibility of recovery	P12: ‘My wish was that it would become easier to talk about it and that I would become better at managing it’. P2: ‘I actually wanted to be pushed a little more. I think that's a way of breaking down barriers.’	None	Participants described participation as creating confidence that change was possible and that they could develop new ways of managing everyday challenges.	Hope for change Overcoming barriers Belief in recovery	Peer discussions instilled hope by normalizing participants' experiences and strengthening their belief that recovery and meaningful change were possible.	Hope was experienced as an evolving recovery process in which peer interactions played a central role. Participants described how sharing experiences with others who had faced similar challenges normalized their own experiences and made recovery seem attainable. This fostered motivation to pursue change, increased confidence through recognition of personal progress, and inspired new goals and aspirations. Consequently, the different dimensions of hope were closely entangled and should be understood as interacting processes rather than distinct categories.
	Hope‐inspiring relationships	P7 ‘It's about how we support one another and say, That's not unusual—I feel that way too…….It made me realize that I'm not the only one who feels this way. Other people have similar experiences, and that was comforting.’	None	Mutual support and recognition of shared experiences reduced feelings of isolation and fostered reassurance and hope.	Hope through mutual support	Relationships with peers who shared similar experiences fostered hope by normalizing participants' struggles.	
	Motivation to change	P6: ‘It's kind of exciting to try and gain a bit of insight into myself. And it helps keep me motivated, you know?’ P4: ‘You have to create new habits. It doesn't help to keep doing what you did before.’	None	Increased self‐understanding encouraged participants to continue working on personal goals and lifestyle changes.	Motivation for change Self‐awareness Behavioural change	Greater insight into oneself fostered optimism and strengthened motivation to engage in recovery‐oriented behaviours.	
	Positive thinking and valuing success	P1: ‘My self‐esteem has become a little better after being in the group’. P10: ‘You have to be happy with what you can do, rather than sad about what you can't.’	P4: ‘I believe in myself more and more now. I've been sober for four months’.	Participants recognized personal achievements and developing strengths, reinforcing confidence in future progress.	Improved self‐confidence Recognizing progress Positive self‐evaluation	Acknowledging progress and personal strengths strengthened hope and confidence in maintaining recovery.	
	Having dream and aspirations		P2: ‘Another group member talked about to start further education. I learned something from that – a person with a mental illness can pursue an education, which I also want to do now. (…) I have become more positive and a bit more constructive about my future.’	Seeing peers pursue dreams and goals inspired participants to believe that similar opportunities were possible for themselves and increased optimism about the future.	Hope through peer role modelling	Observing peers achieve personally meaningful goals transformed participants' expectations of their own future.	

Subsequently, patterns of meaning were examined across the quotes within each category. Relevant quotes were compiled to form a comprehensive representation of each category. The analytical descriptions for each category were refined through iterative revisions. The presentation of results was structured around the five CHIME domains, incorporating contextualized quotes drawn from the entire dataset. Each domain was further divided into findings from the end of PCGI interviews and the follow up interviews to illustrate how the PCGI influenced participants' recovery process. Any disagreements during the coding process were resolved through dialogue in the research team. The research team contributed to all phases of the analysis. See Table 2 for CHIME domains and subcategories and illustrative quotes. Trustworthiness was addressed through attention to credibility and reflexivity. Credibility was strengthened through systematic analysis and ongoing discussions within the research team, as well as by presenting illustrative participant quotes to support the findings. Reflexivity was maintained through continuous reflection on the researchers' preunderstanding and potential influence on data interpretation.

**TABLE 2:** Illustrations from the analysis process and examples of selected quotes.

CHIME	Quotes	
Categories and subcategories	Post intervention	3 months follow‐up
**Connectedness** *Peer support and support groups, Relationships, Support from others, Being part of the community*	‘You also learn how others feel about it, their perspectives. Like, what's the word? There are lots of words of wisdom you can take away and learn from yourself.’ (P13)	‘Just sitting there and hearing that I'm not the only one who's been through something like that? And then it hits you, there are actually a lot of people who've had difficult experiences like that.’ (P6)
**Hope and optimism about the future** *Belief in possibility of recovery, Hope‐inspiring relationships, Motivation to change, Positive thinking and valuing success, Having dreams and aspirations*	‘It's kind of exciting to try and gain a bit of insight into myself. And it helps keep me motivated, you know?’ (P6)	‘And I kind of hoped that there would be some more initiatives (…) You're sort of waiting to see what happens, I think. And I would actually like to work more with my problems.’ (P9)
**Identity** *Dimensions of identity, Rebuilding/redefining, Overcoming stigma*	‘I think about how I need to remember to say no and so on. To set boundaries. To remember myself and check in with what I want, because I always just think about what others want and that kind of thing.’ (P7)	‘I've gained more self‐worth and confidence not that I'm finished with that development, but it's given me a boost, it made me stronger.’ (P5)
**Meaning in life** *Meaning of mental illness experiences, Spirituality, Quality of life, Meaningful life and social roles, Meaningful life and social goals, Rebuilding life*	‘I think I've gotten better at identifying the things that affect me mentally. It used to be hard for me to pinpoint exactly what it is that's affecting me, what's making me feel bad, what's causing this or that in relation to my mental illness.’(P16)	‘It has helped me understand my situation better. I mean, it has made things a bit easier for me.’ (P9)
**Empowerment** Personal responsibility, control over life, focusing upon strengths	‘Yeah, I mean, there are probably some things now that I feel surer about and that I stand by. Whereas before, I could probably be easily manipulated into just doing things.’ (P11)	‘I have become more aware of my challenges, limitations and strengths, so I can more quickly come to terms with what I need to talk about. Previously, I would show up and wait for her to ask me questions. Now I bring up what I want to talk about and need help with.’ (P5)

### 3.9 Ethical Considerations

Participants were informed that participation was voluntary and that they could withdraw at any time without consequences. To protect participant anonymity, findings are presented at the group level, except where individual quotations are included. The regional research administration was notified about the study, and the study was registered as part of North Denmark Region's record of processing activities (Project ID: F2019‐8). According to existing legislation and guidance from the Danish National Centre for Ethics, qualitative health research, such as studies based on individual interviews, does not require formal ethical approval (Ministry of The Interior and Health 2020).

## 4 Results

The analysis revealed that participation in the PCGI had a significant impact on participants' personal recovery process across all categories of the CHIME conceptual framework, both immediately after the intervention and at the three‐month follow‐up.

### 4.1 Connectedness

After participating in the PCGI, all participants emphasized that being among like‐minded peers was a positive and meaningful experience, contributing to a sense of belonging and mutual trust. The realization that others encountered similar problems in daily life with mental illness, combined with the sharing of personal experiences and management strategies, was particularly appreciated and fostered a safe, supportive atmosphere. Some participants further noted that they were inspired to adopt others' management approaches in their own lives.

I think we had such good talks, and it was actually very nice, instead of always talking to someone who is smart and educated. It was nice to talk to someone who had experienced mental illness and been in the same system. (P8)

The safe space in the group was defined by mutual tolerance, acceptance and understanding among participants. Numerous participants described the process of learning from one another as reciprocal, encompassing both the offering and receiving of advice or management strategies. Importantly, some participants noted that contributing solutions to others' challenges enhanced their own positive sense of self.

We have given each other a lot, because people were very open and talked very well to each other and properly together. So, there was respect in the group, and we could give each other advice and guidance. (P10)

Some participants described that the sense of connection established within the group enhanced their comfort and confidence in social interactions beyond the group context. A few further noted that the experience of identifying and articulating personal difficulties during group sessions facilitated their ability to communicate similarly in other relationships. Correspondingly, several participants expressed that engagement in the group had a positive impact on their self‐esteem.

A few participants mentioned that the positive relationships formed within the group extended beyond the group sessions, as reflected in the emergence of friendships among some members.

I think we've made some good connections. We have people we can turn to if we're having a bad day, so it has really brought something positive. (P8)

Additionally, some participants described having a community that extended beyond the group sessions. For example, some travelled together by public transportation to and from the sessions, some arrived early or stayed afterward to talk, and some maintained contact through a group chat on social media. These interactions served to support one another when needed, continue discussions from the sessions, or simply share everyday experiences.

Three months after participating in the PCGI, nearly all participants reiterated the importance of interpersonal relationships, emphasizing that positive social connections continued to play a vital role in enhancing their overall well‐being in everyday life.

I think I've become more open, more relaxed (…) I've become that way even when I've been around other people, or strangers, or people you know or don't know. (P2, follow‐up)

Participants described becoming more open‐minded and trusting in their relationships, both within mental health services and in their everyday lives, emphasizing that the development of social competence was an ongoing process.

### 4.2 Hope and Optimism About the Future

After participating in the PCGI, all participants stated that their involvement in the group fostered a deeper self‐understanding and inspired ‘wishes for a better future’. Sharing and learning from others' experiences were particularly emphasized as hope‐inspiring and empowering aspects of the process. Most participants described this renewed sense of optimism as motivating factor to confront everyday challenges. Additionally, some became aware of unhelpful and unhealthy habits that they wished to change into healthier ones. For instance, during their participation in the PCGI, some participants with alcohol dependency reported reducing or completely stopping their alcohol consumption.

Because there are other things that have changed as well, it's not just that I'm staying away from alcohol. For example, I've really gotten into exercising, which I've done before, but now I've gotten properly into it, and that definitely improves my mood, doesn't it? (P14)

A few participants highlighted that acknowledgment of their progress by family members enhanced their motivation to pursue change and address challenges in daily life.

Three months after participating in the PCGI, participants continued to view their encounters with peers as a source of hope and inspiration. Most participants emphasized that the group's work on identifying personal challenges had strengthened their optimism about the future. Some further described hope as a motivating force, expressing renewed belief in the potential for positive developments in areas such as education and romantic relationships.

Another group member talked about to start further education. I learned something from that – a person with a mental illness can pursue an education, which I also want to do now. (…) I have become more positive and a bit more constructive about my future. (P2, follow‐up)

However, one participant emphasized that sustaining their level of hope following the PCGI would depend on follow‐up support from mental health services to help address the challenges identified during the group.

### 4.3 Identity

After participating in the PCGI, most participants reported that engagement in the PCGI alongside peers had a positive impact on their self‐perception and contributed to a broader self‐understanding. For some, this process fostered increased self‐esteem and confidence, whereas others acknowledged that further development of these attributes remained necessary.

It gives me confidence and self‐esteem, and even more, you know? All of a sudden, I actually believe in myself—I've never done that before, never. (P4)

Prior to participating in the PCGI, some participants shared having spent much of their lives preoccupied with others' opinions of them. Others described constantly questioning themselves ‘am I good enough’ and questioning their adequacy and striving to meet societal expectations while setting unhelpful boundaries for themselves. All participants noted that this negative self‐perception changed following participation in the PCGI. Peer relationships and the emphasis on problem‐solving were particularly highlighted as key factors contributing to improvements in self‐esteem and self‐confidence, enabling participants to view themselves in a more positive and accepting light.

And also accepting myself, that I am good enough, even though I don't have a full‐time job and 4 kids. (P10)

I have learned a lot about myself when we talked about whether the past has influenced me. There are things I hadn't considered that could have affected my life. You go a bit deeper, and you realize things about yourself because you look at it in a different way. (P16)

Furthermore, some participants specified that developing a more positive sense of self and greater self‐acceptance enabled them to be more open about their mental illness in everyday life.

Three months after participating in the PCGI, the enhanced positive sense of self appeared to be maintained as reflected in the participants' interview responses. Most described feeling more confident, resilient and optimistic about themselves. Some specifically mentioned experiencing a greater sense of empowerment, noting a shift in focus toward their lives and personal identities rather than defining themselves solely through their mental illness.

I am more relaxed; I have become a little more forgiving towards myself and my illness. In other words, I have come to accept it a bit more. (P9, follow‐up)

The process of redefining oneself with a more positive mindset contributed to an enhanced sense of self‐worth and fostered feelings of equality with others in society. Participants collectively viewed the redefinition of a positive identity as a continuous process that was initiated during their participation in the PCGI group.

### 4.4 Meaning in Life

After participating in the PCGI, all participants indicated that engagement in the group promoted a deeper awareness of the factors influencing their mental well‐being in daily life. Some participants described developing a new meaning and understanding of their symptoms, which enabled them to develop new strategies for managing illness‐related problems and empowered them to take action in their lives.

I had an expectation that we would just sit and talk together, but Guided Self‐Determination supported that you had to talk about the central things and what was important. (P3)

All participants described engaging in purposeful, goal‐oriented efforts to address the challenges identified and selected during the group sessions. The most frequently mentioned issues concerned difficulties in social relationships, efforts to reduce or eliminate excessive alcohol use, and struggles with self‐worth and confidence in personal abilities.

Three months after participating in the PCGI, nearly all participants indicated that they maintained engagement in goal‐oriented efforts to address their challenges. An increased awareness of the challenges they faced in everyday life was highlighted as a crucial factor motivating them to persist in these efforts. Some participants emphasized the value of focusing on opportunities instead of limitations and of actively striving to improve their overall quality of life. These goal‐oriented efforts were now primarily focused on strengthening social relationships.

Part of the learning I have gained has come gradually, as I have had to integrate it, try it out, and reflect on what works for me from the things I took away (…). I have developed a much better relationship, especially with my family, because I am now able to set boundaries and express my opinions. (P5, follow‐up)

All participants reported becoming more actively engaged in their social lives, which they described as reinforcing their positive sense of identity and enhancing their overall quality of life. Participants who had previously struggled with excessive alcohol consumption and had stopped drinking during their participation in the PCGI continued to maintain abstinence. According to the majority of participants, their social networks had noticed and acknowledged the positive changes in their daily lives.

### 4.5 Empowerment

After participating in the PCGI, all participants reported a significant improvement in their ability to manage their daily lives. Increased self‐insight enabled them to identify new strategies and to feel more confident in addressing and overcoming challenges.

I have developed more in such a short time, than I have in the past. I have gained several really valuable insights, which I have already incorporated into my everyday life. (P5)

Most participants described engaging in a process of self‐reflection, during which they examined their past behaviours and thought patterns. This process facilitated the development of improved strategies for balancing resources and daily obligations, ultimately contributing to prevent the exacerbation of their mental illness.

I am more able to recognize when I need to set boundaries, go home to clear my mind, or retreat to a room by myself before it becomes too overwhelming. (P15)

Some participants particularly emphasized that increased self‐esteem contributed to a stronger sense of control over their lives. They described this as improvements in their *ability to assert themselves*, *remain committed to their decisions*, *demonstrate persistence*, and *fulfil responsibilities*, all of which they attributed to an enhanced sense of self‐confidence.

Three months after participating in the PCGI, all participants reported greater acceptance of their challenges and limitations, along with an enhanced sense of responsibility for their own lives. They further acknowledged the interconnection between life and illness, emphasizing the importance of maintaining a balanced perspective that integrates both and supports overall well‐being.

I have accepted certain things, including that it doesn't have to go so fast and that it is something one works on throughout life. (P3, follow‐up)

Overall, participation in the PCGI was perceived as providing practical tools for everyday life, such as *setting boundaries with oneself and others*, *reflecting on personal circumstances*, *making decisions*, and *remaining present in the moment*. These tools were regarded as particularly beneficial. Nonetheless, some participants emphasized the importance of continued professional follow‐up to sustain and effectively apply the progress achieved through the PCGI in the long term.

## 5 Discussion

The results showed that the PCGI supported participants' personal recovery process by fostering self‐awareness, empowerment, and hope through shared peer experiences in a safe and supportive environment. Furthermore, the PCGI enabled lasting improvements in self‐esteem, self‐management strategies and social functioning, supporting participants achieve greater balance, confidence and control in their daily lives with mental health challenges.

The PCGI consisted of two recovery‐oriented elements, two discovery group sessions and 10 GSD sessions. The active component in the discovery sessions is pre‐prepared, simple questions designed to encourage participants to share their thoughts and reflections and to promote connections among group participants (Barker and Buchanan‐Barker 2005). In this study, all participants reported a strong sense of connectedness, emphasizing that sharing experiences during the group sessions was particularly important and meaningful. The discovery group sessions thus appear to constitute a significant foundation for supporting the personal recovery process. This aligns with the experiences reported by the facilitators and participants, who described the discovery group sessions as meaningful, creating a safe atmosphere and trusting relationship among participants and facilitators (Benzon and Jørgensen 2024).

The active component of the GSD method consists of semi‐structured reflection sheets completed by the participant and discussed during group sessions. In the study exploring the implementation outcomes of the PCGI, facilitators and participants alike emphasized that the reflection sheets offered structure, guidance and clearness within the GSD sessions (Jensen et al. 2024). The reflection sheets address three main topics: *your life with an illness*, *between ideal and reality* and *working to change* through identifying challenges, and reflecting on problem solving strategies (Zoffmann 2004). The results of this study suggest that the person‐centred focus of the GSD method supports all five elements in the CHIME framework and promotes participants' personal recovery, supporting the positioning of the GSD method as a recovery‐oriented intervention. The group format further seemed to enhance the promotion of recovery, as participants consistently emphasized the value of being with likeminded, sharing similar problems, learning from one another, and offering mutual support. This is consistent with previous research showing that peer sharing, guidance, and lived experience exchange within group‐based interventions can facilitate recovery (Gillis and Parish 2019). In the study exploring the implementation outcomes of the PCGI, it was evident that the facilitators played a crucial role in facilitation of the group sessions. The conversation activity varied between groups, requiring different facilitation approaches, ranging from directive to supportive and guiding to ensure that all participants were actively engaged in the dialogue (Jensen et al. 2024).

An integrative review by Linnet and Jørgensen (2023) investigating the impact of the GSD method across diagnoses and health care settings found that patients' experiences with GSD emphasized its ability to promote self‐insight into their current situation. This allowed them to distinguish between life and illness, thereby generating person‐specific knowledge that guided person‐centred support. Participants also highlighted that close collaboration with health care professionals made them feel acknowledged and part of a trusting relationship in which the exchange of knowledge facilitated the development of life skills (Linnet and Jørgensen 2023). In the present study, however, participants primarily emphasized the value of peer relationships and the reflection sheets as key factors influencing their personal recovery process. This does not imply that facilitators were unimportant, as emphasized in the implementation outcomes study, but rather that participants may have been less aware of their role in the group setting compared to individual delivery of the GSD method, as described in the review by Linnet and Jørgensen (2023).

In our study, some of the participants emphasized the importance of professional follow‐up after the PCGI to sustain and further develop the benefits achieved. The literature similarly highlights the value of booster sessions and follow‐up support in maintaining intervention effects. In relapse prevention for individuals with depression, however, it has been discussed whether everyone benefits equally, as those most likely to engage in booster sessions are often those who need them least, suggesting that follow‐up support should be individualized rather than implemented as a ‘one‐size‐fits‐all’ approach (Hlynsson et al. 2025).

The study has several strengths and limitations. A key strength is the longitudinal qualitative design, which enabled exploration of the participants' recovery experiences both immediately after the PCGI and three months later, providing valuable insights into the short‐term impact of the PCGI on personal recovery. However, the study is limited by the short follow‐up period of three months, which may not fully capture long‐term impact on personal recovery. Additionally, only seven of the original 17 participants took part in the follow‐up interviews, which may have influenced the representativeness of the findings. Future research should include a longer follow‐up period and a larger sample to explore the sustainability and broader applicability of PCGI's impact on personal recovery.

The use of the CHIME framework further strengthened the analytical rigour and ensured a structured approach to interpretation of the recovery process. A key strength of this framework is its transdiagnostic applicability, which is particularly relevant as participants in this study included individuals with psychotic or affective disorders, as well as some with alcohol dependency. Furthermore, both the discovery group and the GSD method are designed to be applicable across conditions and diagnostic categories. Consequently, a major strength of this study lies in demonstrating that the PCGI appears to be supporting the recovery process regardless of diagnosis.

## 6 Conclusion

The PCGI supported participants' recovery by enhancing self‐awareness, self‐esteem and hope for the future. Through shared experiences in a safe and supportive peer environment, participants developed new coping strategies, improved social functioning, and gained a stronger sense of identity and empowerment. They reported greater acceptance of themselves and their challenges, as well as sustained positive changes in daily life and relationships. Overall, the PCGI fostered personal growth, connection, and a renewed sense of control in the participants' recovery processes. However, it was also emphasized that continued professional follow‐up was important to sustain and effectively implement these gains in the long term.

### 6.1 Relevance for Clinical Practice

Our results suggest that the discovery group and GSD sessions play an important role in improving participants' personal recovery within the PCGI. Although participants attributed greater importance to peer relationships and the reflection sheets than to the facilitators, it remains essential that facilitators possess the necessary skills and knowledge of the PCGI content, effectively manage group processes, and apply recovery‐oriented practices. Furthermore, facilitators should have access to supervision to support their role and professional development.

## Author Contributions

R.J. designed the study. K.L.H., K.J., F.M.M. and R.J. participated in analysing the data and writing the first draft of the manuscript. K.L.H., K.J. and R.J. participated in revising the first draft. All authors participated in revising the following drafts and approved the final version.

## Funding

The authors have nothing to report.

## Consent

All participants provided written informed consent to take part in the study, including consent for the collected data to be published in a scientific journal and presented at conferences.

## Conflicts of Interest

The authors declare no conflicts of interest.

## Data Availability

Research data are not shared.
